# Elevated *Plasmodium* Sporozoite Infection Rates in Primary and Secondary Malaria Vectors in *Anopheles stephensi*-Infested Areas of Ethiopia

**DOI:** 10.3390/insects16050462

**Published:** 2025-04-27

**Authors:** Dawit Hawaria, Timotwos Amanuel, Abraham Anbesie, Daibin Zhong, Solomon Kibret, Ming-Chieh Lee, Guofa Zhou, Chloe Wang, Jiale Chen, Tafesse Matewos, Amanuel Ejeso, Chirotaw Ayele, Temesgen Yosef, Delenesaw Yewhalaw, Guiyun Yan

**Affiliations:** 1 School of Public Health, Hawassa University, Hawassa P.O. Box 05, Ethiopia; tafessemk@gmail.com (T.M.); aejeso@yahoo.com (A.E.); chirotawa@hu.edu.et (C.A.); temesgenyoseph7@gmail.com (T.Y.); 2 School of Public Health, Wolaita Sodo University, Sodo P.O. Box 138, Ethiopia; timotwosamanuel@gmail.com; 3 Department of Public Health, Arbaminch Health Science College, Arba Minch P.O. Box 55, Ethiopia; abrishanbesie03@gmail.com; 4 Program in Public Health, University of California at Irvine, Irvine, CA 92697, USA; dzhong@hs.uci.edu (D.Z.); mingchil@uci.edu (M.-C.L.); zhoug@hs.uci.edu (G.Z.); xiaomiw1@hs.uci.edu (C.W.); jialechen873@ucsb.edu (J.C.); guiyuny@hs.uci.edu (G.Y.); 5 West Valley Mosquito and Vector Control District, Ontario, CA 91761, USA; s.kibret@gmail.com; 6 Tropical and Infectious Diseases Research Centre (TIDRC), Jimma University, Jimma P.O. Box 378, Ethiopia; delenasawye@yahoo.com

**Keywords:** *Anopheles stephensi*, primary *vector*, secondary vector, sporozoite, bloodmeal, mosquito control, Ethiopia

## Abstract

Assessing bloodmeal sources and sporozoite infection rates in mosquito vectors is essential for understanding their role in malaria transmission. This study investigated these factors in Hawassa, Southern Ethiopia—an area recently invaded by *Anopheles stephensi*—through adult mosquito collections conducted between January and April 2023 using BG-Pro traps, CDC Light Traps, and Prokopack Aspirators. A total of 738 female *Anopheles* mosquitoes were collected, including *An. arabiensis* (72.9%), *An. pharoensis* (13.4%), *An. stephensi* (7.5%), and *An. coustani* (6.2%). Human Blood Index (HBI) values were 23.3% for *An. arabiensis*, 43.8% for *An. pharoensis*, 8.3% for *An. stephensi*, and 25.0% for *An. coustani*. *Plasmodium* infection was detected in 8% of *An. arabiensis* and 4.7% of *An. pharoensis*, while *An. stephensi* and *An. coustani* were uninfected. The findings highlight the presence of multiple competent malaria vectors, with significant infection rates observed in both primary and secondary species, underscoring the need to include secondary vectors in control and surveillance programs. The detection of *An. stephensi* alongside native vectors adds complexity to malaria control efforts, reinforcing the importance of continuous monitoring to understand the changing dynamics of vector populations and their roles in transmission.

## Simple Summary

This study investigated malaria transmission by analyzing sporozoite infection rates and bloodmeal sources in adult mosquitoes. Multiple efficient malaria vectors were identified, including the invasive *Anopheles stephensi*. High *Plasmodium* infection rates were found in both primary and secondary mosquito vectors, emphasizing the need to include secondary vectors in surveillance and control efforts. Although *An. stephensi* tested negative for malaria sporozoite infection, its presence complicates malaria control. The findings stress the importance of ongoing monitoring to better understand the roles of various mosquito species in transmission and to strengthen vector control strategies.

## 1. Introduction

Understanding vector dynamics in disease transmission is crucial for designing effective vector control interventions against malaria. In Ethiopia, about 47 *Anopheles* species have been documented [1,2]. The primary vector of malaria is the *Anopheles arabiensis*, while *An. pharoensis, An. funestus, An. coustani* complex, and *An. nili* play secondary roles [2]. Recently, the Asian malaria vector *Anopheles stephensi*, a competent vector of both *P. falciparum* and *P. vivax*, invaded the East African region [3,4,5], complicating the existing malaria control program.

Different malaria vector species exhibit varying bloodmeal preferences, influenced by host availability. Some are anthropophilic (preferring humans), while others are zoophilic (preferring animals) or opportunistic (preferring both) [6,7,8,9,10,11]. These feeding preferences play a critical role in malaria transmission dynamics. Anthropophilic vectors present the highest risk because of their tendency to frequently feed on humans. Both *An. arabiensis* and *An. pharoensis* are considered an opportunistic feeder in Ethiopia [12,13,14,15]. *Anopheles stephensi*, on the other hand, is predominantly documented to have zoophilic behavior [16,17].

In Africa, malaria transmission is facilitated by multiple *Anopheles* species, each exhibiting different vectorial capacities and behaviors [6]. In recent years, changes in vector dynamics have been documented in South and East Africa. For instance, Mustapha et al. (2021) [18] indicated that secondary vectors may contribute substantially to malaria transmission because of their high densities and *Plasmodium*-positivity rate in Kenya [18]. This demonstrates that in settings with multiple malaria vectors, continuous monitoring of their vectorial role is essential to understand the role of diverse vector species in malaria transmission and design control measures accordingly.

Hawassa City, the largest city in Southern Ethiopia, is endemic to malaria with both *P. falciparum* and *P. vivax* prevalent [19,20]. The city has recently experienced an increase in malaria cases and incidence to an epidemic level. Multiple malaria vectors have been reported in the area, including *An*. *stephensi*, *An. arabiensis*, *An. pharoensis*, and *An. coustani* [19]. However, the role of diverse vector species in local malaria transmission has not yet been defined.

This study aims to identify the bloodmeal sources and sporozoite infection rates of *Anopheles* mosquitoes in Hawassa City, Ethiopia. Better understanding of vectorial characteristics helps guide the development of comprehensive control strategies and prevent recurrent outbreaks.

## 2. Methods

### 2.1. Study Design and Period

**Figure 1:**
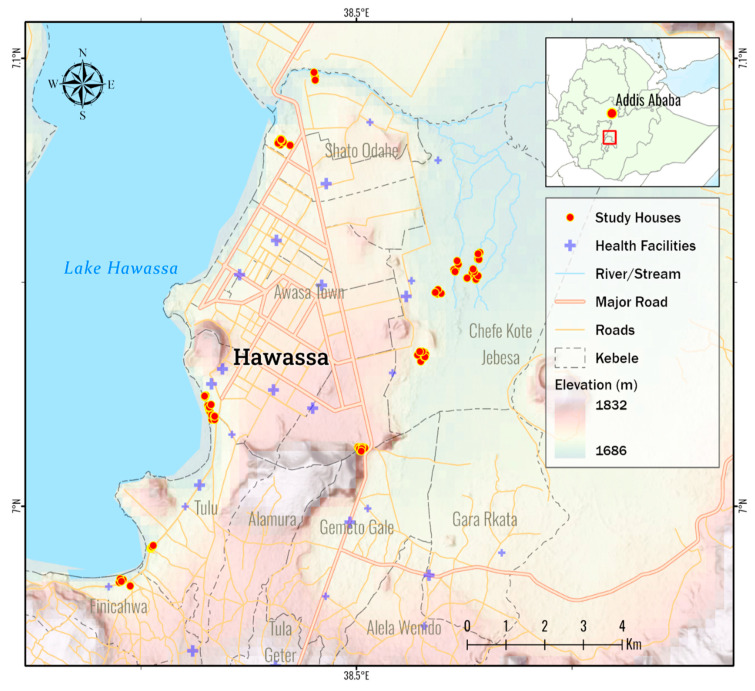
Map of the study setting, Hawassa City, Ethiopia.

A targeted entomological survey was conducted from January 2023 to April 2023 in Hawassa City, 275 km from the Capital, Addis Ababa, in Southern Ethiopia (Figure 1). The Study setting has been described elsewhere [19]. For vector surveillance, malarious villages (*kebeles*) were selected based on the report by the Hawassa City’s Health Department [20].

### 2.2. Mosquito Survey

Three mosquito surveillance tools were employed to collect adult mosquitoes: CDC Light Traps (Model: John W. Hock CDC Light Trap 512, Gainesville, FL, USA); Bioagent (BG-pro) Traps with attractant lure (Biogents AG, Regensburg, Germany); and Prokopack Aspirator (John W. Hock 1418, Gainesville, FL, USA).

Indoor mosquito collections with CDC Light Trap and BG-Pro were conducted overnight from 18:00 to 06:00, with traps suspended 1.5 m above the ground near sleeping areas. For outdoor sampling, traps were set at least 5 m away from houses. The following morning, the trap collection boxes were gathered and transported to the Malaria Research Laboratory at Hawassa University for the retrieval of mosquitoes and specimen processing. A total of 180 and 120 trap-nights were set with BG-Pro and CDC Light traps, respectively. In addition, Prokopack Aspirator was used to sample indoor resting mosquitoes in the morning between 6:30 and 8:00 in 60 randomly selected houses.

### 2.3. Mosquito Specimen Processing

Collected mosquito specimens were brought to the Malaria Research Laboratory at Hawassa University for species identification and abdominal status check. Live mosquitoes were euthanized using chloroform. The mosquitoes were then emptied into a Petri dish and sorted into culicines and anophelines. Culicines were counted, recorded, and discarded, while all *Anopheles* mosquitoes were further sorted into species using a morphological key [21]. The abdominal status of each specimen was also assessed using a stereomicroscope and categorized as unfed, fed, half-gravid, or gravid. Each female *Anopheles* mosquito was individually kept in labeled Eppendorf tubes for further molecular analysis. Each tube was labeled with the collection date, abdominal status, trapping method, trapping location, morphological identification number, and species.

### 2.4. Mosquito DNA Extraction

The head-thoracic part of each female adult mosquito was dissected for DNA extraction. DNA extraction was performed following the established automated DNA extraction protocol outlined by Zhong et al. [22]. Briefly, DNA extraction utilized the QIAamp 96 DNA QIAcube HT kit with a QIAcube HT 96 automated nucleic acid purification robot (Qiagen, Valencia, CA, USA), following the manufacturer’s protocol with minor modifications. Specifically, each sample was ground in a ZR Bashing Bead Lysis tube (2.0 mm beads, Zymo Research Corporation, Irvine, CA, USA) and homogenized in 200 μL of lysis solution containing 20 µL of proteinase K (20 mg/mL) using the TissueLyser II system (Qiagen, Hilden, Germany) for 10 min at 30 Hz. The genomic DNA was eluted in a final volume of 100 µL for the head-thoracic portion and 200 µL for the abdominal portion. The DNA extracted from the head–thoracic portion was used for detecting *Plasmodium* sporozoites, while the DNA from the abdominal portion was used for species and bloodmeal identification [22].

### 2.5. Molecular Genotyping of Species, Bloodmeal, and Parasite Infections

For confirmation, *An. stephensi* and *An. arabiensis* was screened by PCR using a previously established protocol [19,23]. PCR reactions were carried out in a total volume of 17 μL, containing 1 μL of DNA template, 5 pmol of each primer, and 8.5 μL of DreamTaq Green PCR Master Mix (2×) (Thermo Fisher Scientific, Waltham, MA, USA). The thermocycling protocol involved an initial activation step of 3 min at 95 °C, followed by 35 amplification cycles of 30 s at 94 °C, 30 s at 55 °C, and 45 s at 72 °C, with a final extension step of 6 min at 72 °C. Other *Anopheles* species were identified through DNA sequencing of the ITS2 region of nuclear ribosomal DNA, following the methods described by Zhong et al. [22].

Mosquito bloodmeal sources (humans, cows, pigs, goats, and dogs) were examined using qPCR using previously established protocols [23,24,25]. *Plasmodium* infections (*Plasmodium falciparum*, *Plasmodium vivax*, *Plasmodium malariae*, and *Plasmodium ovale*) in mosquitoes were examined by qPCR detection using established protocols [26,27,28]. Multiplexed qPCR was performed on a QuantStudio 3 Real-Time PCR System (Thermo Fisher Scientific, Carlsbad, CA, USA) in a final volume of 12 µL, including 2 µL of sample DNA, 6 µL of PerfeCTa qPCR ToughMix, Low ROX Master Mix (2×) (Quantabio, Beverly, MA, USA), 0.5 µL of each probe (2 µM), and 0.4 µL of each forward primer (10 µM) and reverse primer (10 µM). Positive and negative controls were used in all qPCR runs. Positive controls were DNA from *P. falciparum* 3D7 (MRA-102G), *P. vivax* Sal I (MRA-46G), *P. malariae* 18S (MRA-179), and *P. ovale* 18S (MRA-180) (all from BEI Resources). Molecular-grade water and uninfected sample DNA served as negative controls. To minimize issues with PCR inhibition, we used the PerfeCTa qPCR ToughMix, noted for its inhibitor resistance, and visually screened amplification curves for signs of inhibition like abnormal shapes or low plateaus. The temperature profile involved a hold stage at 50 °C for 2 min and 95 °C for 2 min, followed by 45 cycles of PCR amplification at 95 °C for 3 s and 60 °C for 30 s.

### 2.6. Data Analysis

Mosquito density was calculated as the mean number of mosquitoes caught per trap—night for each trap type. *Anopheles* species density difference across traps was compared using independent Samples Kruskal-Wallis Test. *Plasmodium* sporozoite infection rate was calculated as the proportion of mosquitoes infected with *Plasmodium* against those tested. The Entomological Inoculation Rate (EIR) was estimated by multiplying the sporozoite rate by the man-biting rate. The man-biting rate can be determined directly if mosquito collection is conducted through human landing catches (HLC). However, since mosquito collection in this study was done using traps, the man-biting rate was estimated by dividing the mosquito density obtained from trap catches by a conversion factor of 1.605 [29]. The human blood index (HBI) and bovine blood index (BBI) were calculated as the proportion of fed mosquitoes that fed on human and bovine bloodmeals, respectively [8,30,31]. Mixed bloodmeals (human + bovine) were included in the counts for both HBI and BBI. All data was analysed using Microsoft Excel (Version 2016, Microsoft Corp, Redmond, WA, USA) and IBM SPSS version 25.0 (SPSS Inc., Chicago, IL, USA).

### 2.7. Ethical Considerations

The study protocol was approved by the Institutional Review Board (IRB) of the College of Medicine and Health Sciences, Hawassa University (Ref. No: IRB/069/16). Mosquito collections from each household were conducted after obtaining verbal consent from the household head.

## 3. Results

### 3.1. Anopheles Species and Density

**Figure 2:**
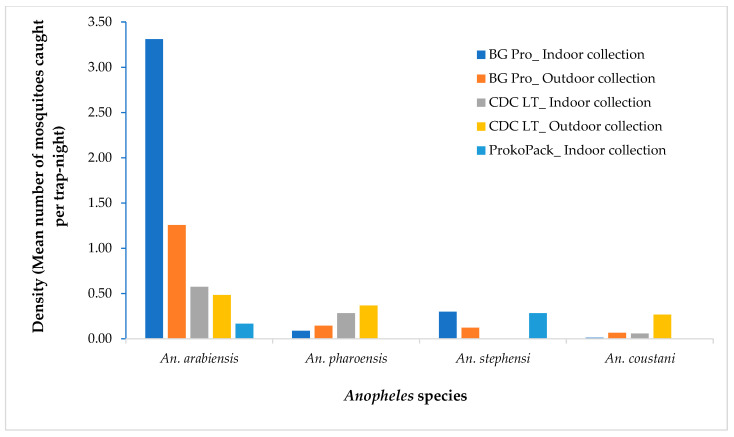
Density of *Anopheles* mosquitoes in various trapping devices in Hawassa, southern Ethiopia, 2023.

A total of 738 female *Anopheles* mosquitoes were collected during the study period. *Anopheles arabiensis* was the predominant species, accounting for 72.9% (*n* = 538) of the collections, followed by *An. pharoensis*, 13.4% (*n* = 99), *An. stephensi*, 7.5% (*n* = 55), and *An. coustani* 6.2% (*n* = 46). A higher density of *An. arabiensis* was recorded in the BG-Pro Trap collection compared to other trapping techniques (df = 2; *p* = 0.004), while a higher density of *An. pharoensis* was observed in CDC Light Trap collection (*p* = 0.006). *Anopheles stephensi* was exclusively captured by the BG Pro Trap and the ProkoPack Aspirator in a comparable density (*p* = 0.254). In both the BG Pro and CDC LT collections, more *An. arabiensis* was collected indoors than outdoors. In contrast, for *An. pharoensis*, outdoor density exceeded indoor density by 1.5 times in the BG Pro collection and by 1.3 times in the CDC collection (Figure 2).

### 3.2. Bloodmeal Source

A total of 456 *Anopheles* mosquitoes were tested for bloodmeal sources. The bloodmeal sources identified included human, cow, goat, and dog (Table 1). Some (37.5%) of the samples were unidentified.

**Figure 3:**
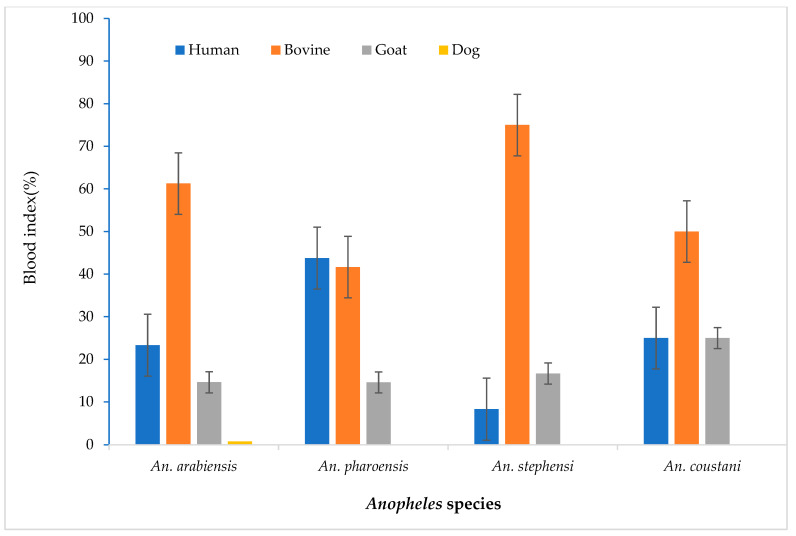
*Anopheles* species bloodmeal index in Hawassa, Southern Ethiopia, 2023.

*Anopheles arabiensis* and *An. stephensi* had lower Human Blood Index (HBI) values, 23.3% and 8.3%, respectively. These species, however, demonstrated a higher Bovine Blood Index (BBI), 61.3% and 75.0%, respectively. *An. pharoensis* showed similar HBI (43.8%) and BBI (41.7%) values (Figure 3).

### 3.3. Sporozoite Infection and Entomological Inoculation Rates

The PCR results showed 4.0% of *Plasmodium falciparum* and 4.0% of *Plasmodium vivax* sporozoite infection in *An. arabiensis*. Likewise, 3.5% of *P. vivax* and 1.2% of *P. falciparum* was detected in *An. pharoensis*, while no infection was observed in both *An. stephensi* and *An. coustani* (Table 2).

The EIR estimated for the study period indicated that *An. arabiensis* contributed 4.50 *P. falciparum*-infective bites and 4.50 *P. vivax*-infective bites per night in the study area. *Anopheles pharoensis* accounted for 0.25 *P. falciparum*-infective bites and 0.72 *P. vivax*-infective bites per night.

## 4. Discussion

The present study highlighted an elevated *plasmodium* sporozoite infection rate in primary and secondary malaria vectors of Ethiopia compared to previous reports. The *Plasmodium* sporozoite infection rate of *An. arabiensis* reported was about seven times higher than in previous studies in Central and Southern Ethiopia [8,32,33,34,35,36]. Similarly, the observed sporozoite rate in *An. pharoensis* was about six times higher than previously reported [32,33,36]. A recent study also documented the increase in malaria incidence in the study area [19], which might be attributed to the high sporozoite infection rate observed in this study in both primary and secondary vectors. This is mainly because the area is prone to malaria epidemics due to the presence of mosquito-breeding lake shorelines of Lake Hawassa coupled with swampy areas surrounding the city. The present findings suggest the need for inclusive interventions considering both primary and secondary vectors for successful control of malaria in the area. Current control measures predominantly target the primary vectors. However, it is noted that the secondary vectors, such as *An. pharoensis* and *An. coustani*, could contribute to residual transmission [6]. This suggests that in regions with multiple efficient malaria vectors, interventions targeting a primary vector alone may not effectively reduce malaria transmission, as other vectors could keep augmenting the transmission. Additionally, potential vector shift may also occur if targeted intervention suppresses the primary vector population, as noted by Msugupakulya et al. (2023) in East and Southern Africa [37].

Malaria transmission in Ethiopia is a complex and dynamic issue, with a substantial gap in understanding the bionomics and role of secondary vectors in disease transmission. *Anopheles pharoensis* was previously incriminated as the second most abundant malaria vector and exhibited the second-highest *Plasmodium* infection rate after *An. arabiensis* in this study, suggesting its significant role in local malaria transmission. Several studies also noted that *An. pharoensis* has consistently ranked second in abundance and sporozoite infection rate elsewhere in Ethiopia [32,33,35]. In another laboratory study, *An. pharoensis* demonstrated similar susceptibility with *An. arabiensis* to *P. falciparum* infection [38]. Unfortunately, less attention has been given to the secondary vectors both in research and vector control interventions in Ethiopia. The study emphasizes the need for a comprehensive understanding of secondary vector biology, ecology, behavior, vector competence, and response to existing interventions. As the country embraces malaria elimination, accurate information on the role of multiple vector species on malaria transmission is critical.

The presence of *An. stephensi* could further pose a significant threat to malaria control efforts in Ethiopia [3]. As the species alarmingly spread to several areas in Ethiopia [4,19,39], control measures are still not well incorporated in routine vector control strategies. Few studies from Eastern Ethiopia have linked the recent regional malaria outbreak to *An. stephensi* [40,41]. In this study, this species occurred in sympatry with native vectors with low density in adult collections in Southern Ethiopia. A recent study in the same study area reported high larval density of *An. stephensi* [19]. This indicates the species is well established in Southern Ethiopia. Although no *An. stephensi* specimens tested positive for *Plasmodium* sporozoites in this study—likely due to the small sample size as a result of a lack of optimal vector surveillance tools for adult *An. stephensi* collection—this species poses a potential risk for malaria transmission [4,39]. Therefore, it is advisable to intensify the development of efficient surveillance tools to collect a good sample size of adult *An. stephensi* specimens for testing in order better define its role in malaria transmission in the region. The lower HBI and *Plasmodium* infection rates observed in wild *An. stephensi* populations, both in this study and earlier works from Ethiopia [4,39], might indicate that the mysorensis form of this species, which has zoophilic behavior, is spreading in Ethiopia (unpublished data). Future research should test these hypotheses in the recently introduced regions of Ethiopia.

This study has several limitations that should be taken into account when interpreting the findings. First, this study was conducted during the dry season. Seasonal variation plays a critical role in influencing vector species composition, density, blood-feeding behavior, and infection status. During the dry season, mosquito densities are typically lower, with resilient species persisting in limited habitats. The relatively small mosquito sample size limits the generalizability of the results of vectors bionomics and their role in malaria transmission. In contrast, the rainy season offers abundant breeding sites, resulting in increased density vectors. Sporozoite infection rates also tend to increase in the rainy season, driven by higher mosquito densities. Second, the choice of mosquito trapping methods can substantially affect estimates of vector density, host preference, and infection status. Mosquito trap type, placement, and maintenance can significantly affect surveillance results by introducing species or behavioral biases and impacting data accuracy. Low *Anopheles stephensi* counts in CDC light traps are likely due to the species’ weak attraction to light, as it is not strongly phototropic compared to other mosquitoes. Additionally, *An. stephensi* prefers urban, indoor, and shaded environments, which may be missed by traps placed outdoors or without human-associated cues like CO_2_ or odor. Its peak biting times may not align with trap effectiveness, and more phototactic species may outcompete it in trap captures. Addressing this limitation through standardized or complementary trapping approaches could improve the accuracy of vector surveillance and malaria control strategies. Third, a considerable proportion of mosquito bloodmeal sources remained unidentified. This is likely due, in part, to the exclusion of some common domestic animals—such as chickens and equines—from the bloodmeal analysis, which may have contributed to the high proportion of unidentified hosts.

In addition, the choice of trapping method can influence species composition in mosquito collections, potentially introducing bias in interpreting species abundance and infection rates. In our study, we employed three complementary trapping tools—BG-Pro traps, CDC Light Traps, and Prokopack Aspirators—to increase species diversity in collections and minimize bias introduced by any single method. However, we acknowledge that *An. stephensi* may be underrepresented due to its lower attraction to light-based traps and preference for indoor resting, behaviors not optimally captured by all trap types used. To address the concern regarding infection rate comparisons, we clarify that our analysis of *Plasmodium* infection was not intended to directly compare infection prevalence between species but rather to provide a species-specific snapshot of infection status based on the mosquitoes collected. Nevertheless, we agree that unequal sample sizes can affect interpretation. To improve future analyses, we recommend standardizing infection screening across equal numbers of each species or adjusting for trapping efficiency using known capture biases.

## 5. Conclusions

In summary, this study confirmed the presence of multiple efficient malaria vectors, with *An. arabiensis* and *An. pharoensis* exhibiting high *Plasmodium* infection rates, underscoring the significant role of secondary vectors in malaria transmission and the need to include them in control strategies. The detection of *An. stephensi* alongside these primary and secondary vectors further complicates malaria control efforts, emphasizing the importance of ongoing surveillance to better understand vector bionomics and their evolving roles. Additionally, future longitudinal studies should aim to collect larger mosquito samples to more accurately assess the relative contribution of each vector species to malaria transmission in the region.

## Figures and Tables

**Table 1:** Bloodmeal sources of *Anopheles* species in Hawassa, Southern Ethiopia, 2023.

Host	*An. arabiensisn* (%)	*An. pharoensis* *n* (%)	*An. stephensi* *n* (%)	*An. coustani* *n* (%)
Human	35 (10.6)	14 (16.5)	1 (3.3)	1 (10.0)
Human + cow	18 (5.4)	3 (3.5)	1 (3.3)	1 (10.0)
Human + cow + goat	1 (0.3)	2 (2.3)	-	-
Human + goat	5 (1.5)	2 (2.4)	-	-
Cow	116 (35.0)	12 (14.1)	16 (53.3)	3 (30.0)
Cow + goat	19 (5.7)	3 (3.5)	1 (3.3)	-
Cow + goat + dog	1 (0.3)	-	-	-
Dog	1 (0.3)	-	-	-
Goat	11 (3.3)	13 (15.3)	3 (10.0)	2 (20.0)
Not detected	124 (37.5)	36 (42.4)	8 (26.7)	3 (30.0)
Total	331	85	30	10

**Table 2:** *Plasmodium* sporozoite infection rates of *Anopheles* species in Hawassa, Ethiopia, 2023.

Anopheles Species	Number Tested	Sporozoite Positive n (%)		Parasite Species			
		Indoor Collection	Outdoor Collection	*Plasmodium falciparum*		*Plasmodium vivax*	
				Indoors	Outdoors	Indoors	Outdoors
*An. arabiensis*	331	13 (4.0)	13 (4.0)	7	6	6	7
*An. pharoensis*	85	3 (3.5)	1(1.2)	1	0	2	1
*An. stephensi*	30	0	0	0	0	0	0
*An. coustani*	11	0	0	0	0	0	0

## Data Availability

The original contributions presented in this study are included in the article. Further inquiries can be directed to the corresponding author.
